# First insight into *Mycobacterium tuberculosis *genetic diversity in Paraguay

**DOI:** 10.1186/1471-2180-7-75

**Published:** 2007-08-08

**Authors:** Norma Candia, Beatriz Lopez, Thierry Zozio, Marcela Carrivale, Chyntia Diaz, Graciela Russomando, Nilda J de Romero, Juan C Jara, Lucia Barrera, Nalin Rastogi, Viviana Ritacco

**Affiliations:** 1 Departamento de Biología Molecular, Instituto de Investigaciones en Ciencias de la Salud (IICS), Universidad Nacional de Asunción, Asunción, Paraguay; 2 Servicio de Micobacterias, Instituto Nacional de Enfermedades Infecciosas, ANLIS "Carlos G. Malbran", Buenos Aires, Argentina; 3 Unité de la Tuberculose et des Mycobacteries, Institut Pasteur de Guadeloupe, France; 4 Laboratorio Central de Salud Pública, Asunción, Paraguay; 5 Programa Nacional de Control de la Tuberculosis, Asunción, Paraguay

## Abstract

**Background:**

We present a picture of the biodiversity of *Mycobacterium tuberculosis *in Paraguay, an inland South American country harboring 5 million inhabitants with a tuberculosis notification rate of 38/100,000.

**Results:**

A total of 220 strains collected throughout the country in 2003 were classified by spoligotyping into 79 different patterns. Spoligopatterns of 173 strains matched 51 shared international types (SITs) already present in an updated version of SpolDB4, the global spoligotype database at Pasteur Institute, Guadeloupe. Our study contributed to the database 13 new SITs and 15 orphan spoligopatterns. Frequencies of major *M. tuberculosis *spoligotype lineages in our sample were as follows: Latin-American & Mediterranean (LAM) 52.3%, Haarlem 18.2%, S clade 9.5%, T superfamily 8.6%, X clade 0.9% and Beijing clade 0.5%. Concordant clustering by IS*6110 *restriction fragment length polymorphism (RFLP) and spoligotyping identified transmission in specific settings such as the Tacumbu jail in Asuncion and aboriginal communities in the Chaco. LAM genotypes were ubiquitous and predominated among both RFLP clusters and new patterns, suggesting ongoing transmission and adaptative evolution in Paraguay. We describe a new and successfully evolving clone of the Haarlem 3 sub-lineage, SIT2643, which is thus far restricted to Paraguay. We confirmed its clonality by RFLP and mycobacterial interspersed repetitive unit (MIRU) typing; we named it "Tacumbu" after the jail where it was found to be spreading. One-fifth of the spoligopatterns in our study are rarely or never seen outside Paraguay and one-tenth do not fit within any of the major phylogenetic clades in SpolDB4.

**Conclusion:**

Lineages currently thriving in Paraguay may reflect local host-pathogen adaptation of strains introduced during past migrations from Europe.

## Background

In spite of the wide availability of cost effective interventions for its control, tuberculosis (TB) is still a major global health problem and the leading cause of death from a curable infectious disease [1]. An increasing amount of evidence indicates that *Mycobacterium tuberculosis*' ability to spread varies from strain to strain and that different strains have different geographical and/or host specificities [2,3].

Since the discovery of DNA polymorphisms in *M. tuberculosis*, molecular typing of strains has become invaluable for the study of epidemiology of TB [4]. Restriction fragment length polymorphism typing using the insertion element IS*6110 *as a probe has been applied since the early 1990s and still is the most reliable method for *M. tuberculosis *strain differentiation [5,6]. The use of additional *M. tuberculosis *genotyping approaches further allows investigators not only to document outbreaks and track epidemics locally, but also to gain an insight into the global migration and expansion of strains [7,2]. Several polymerase chain reaction (PCR)-based techniques have been proposed for *M. tuberculosis *strain typing. The most widely used is spoligotyping, which detects presence or absence of 43 short variable spacer sequences interspersed with direct repeats in the Direct Repeat (DR) region of the chromosome [8]. A more recent approach consists of the analysis of polymorphisms in 12 to 24 loci containing variable number of tandem repeats of Mycobacterial Interspersed Repetitive Units (MIRU) [9]. These two PCR-based methods have been adapted for high-throughput genotyping and combined provide the basis for research on evolutionary genetics of *M. tuberculosis *[10]. The information on *M. tuberculosis *population diversity gathered so far in global databases provides a robust platform for research on phylogeny and virulence [11]. Ultimately, this knowledge is contributing to the design of rational measures for the control of TB.

Paraguay is an inland South American country with 5.2 million inhabitants and an area of 400,000 km^2^; it shares borders with Bolivia, Brazil and Argentina [12]. Politically, the country consists of 17 departments, in addition to Asuncion, the capital city. The Paraguay River runs from North to South dividing the territory in two distinct geographical regions with a remarkable difference in population density. The fertile Oriental Region has 160,000 km^2 ^and as many as 31.5 inhabitants per km^2^. To the northwest is the Chaco, an arid region with a surface of 247,000 km^2 ^and only one inhabitant per km^2^; its people are mainly settled in sparse aboriginal communities. A total of 2,116 TB cases were reported countrywide in 2003, yielding a case notification rate of 37.8 cases per 100,000 inhabitants. In absolute numbers, most cases occur in Asuncion and the Central Department, its contiguous densely populated area; both are located in the Oriental Region. In the Oriental Region the incidence mirrors the national rate whereas in the Chaco the rate is 3 to 5 times higher. There is considerable under-reporting of TB in Paraguay; the World Health Organization estimates the true incidence in the country as 50–99 cases per 100,000 [13].

In 2002 the National TB Program implemented a project on anti-TB drug resistance surveillance sponsored by WHO/USAID. At that time, mycobacterial culture was seldom performed in the country and case finding was mainly based on acid fast bacilli smear examination of symptomatic patients. The availability of isolates from this project permitted this first-ever strain typing study in Paraguay. This investigation was undertaken to provide both a nationwide view of the *M. tuberculosis *population structure and a preliminary assessment of the feasibility and usefulness of genotyping for epidemiological purposes in this limited-resource and endemic TB setting.

## Results

### Population characteristics

Of the 220 strains included in the study, 156 (71%) were isolated from male patients. One hundred forty-six patients (66%) were 20 to 50 years old (mean ± SD: 38.5 ± 15.7, range 10–78). Two hundred and sixteen patients (98%) were native-born; one patient was an immigrant from South Korea; the place of birth was not registered for the remaining three patients, but their demographic data suggested Brazilian origin.

Sixty-seven strains (30%) were isolated from patients diagnosed in either Asuncion or the Central Department (hereafter referred to jointly as the metropolitan area); 114 (52%) originated from other areas in the Oriental Region and 39 (18%) were obtained from the sparsely populated Chaco. One hundred and eighty-four strains (84%) were isolated from newly diagnosed patients and the remaining 36 (16%) from patients previously treated for TB. Thirty-three strains were drug resistant, six of which were multidrug resistant (i.e. resistant at least to rifampicin and isoniazid).

### Lineage assignment according to spoligotyping

**Figure 1:**
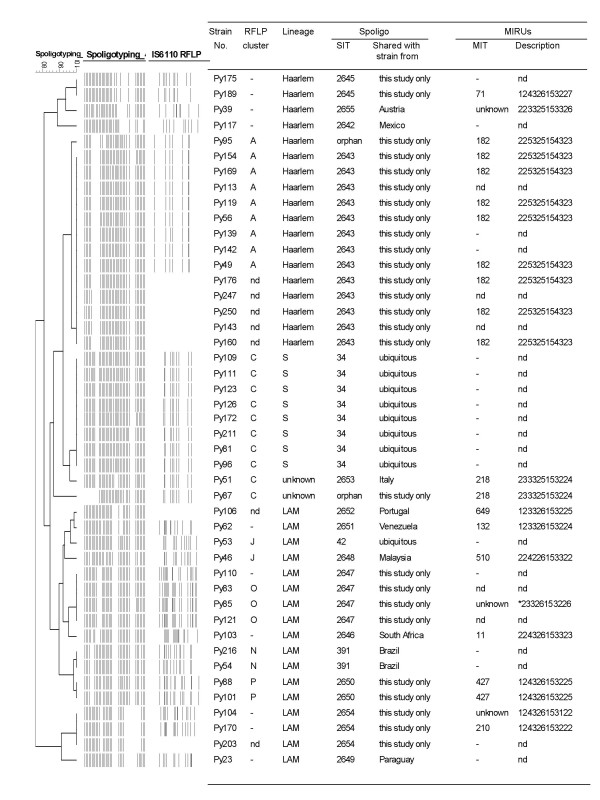
Computer-generated dendrogram according to UPGMA spoligotype analysis and characteristics of selected strains from Paraguay, including strains in 13 newly-created shared spoligotypes absent from DB4 and therefore not mentioned in Table 1 (SITs 2642, 2643, and 2645–2655).

The 220 strains were classified into 79 different spoligopatterns that could be assorted in three groups. The first group included 173 strains matching 51 shared international types (SITs, patterns shared by two or more isolates) already present in an updated version of the SpolDB4 database [14]. For these previously described SITs, Table 1 presents the frequencies found in our study as compared to frequencies in the international database at the time of this analysis, as well as their clade denomination. The second group consisted of 32 strains distributed within 13 newly created SITs, as shown in Figure 1. Five of these new SITs, (2643, 2645, 2647, 2650 and 2654) contained strains only identified in this study. Each of the remaining eight newly created SITs involved a single strain in this study matching with a previously orphan strain in SpolDB4. The third group included the remaining 15 strains that did not match with any other spoligopattern in this study nor in SpolDB4 (Table 2).

**Table 1:** Frequency of 51 shared spoligotypes (SITs) according to Brudey *et al*. [14], identifiedin 173 *Mycobacterium tuberculosis *strains isolated in Paraguay, 2003

SIT	Octal Number	Strains in this study		Strains in SpolDB4		% in this study as compared to SpolDB4	Lineage
				
		n	%	n	%		
42	777777607760771	40	18.2	1926	4.5	2.1	LAM
391	743777607760731	21	9.5	23	0.1	91.3	LAM
34	776377777760771	16	7.3	618	1.4	2.6	S
50	777777777720771	9	4.1	2128	5.0	0.4	Haarlem
93	777737607760771	6	2.7	267	0.6	2.2	LAM
521	777777777760611	6	2.7	22	0.1	27.3	T
52	777777777760731	4	1.8	518	1.2	0.8	T
159	777740017760771	4	1.8	64	0.2	6.3	unknown
4	000000007760771	3	1.4	220	0.5	1.4	unknown
53	777777777760771	3	1.4	3738	8.8	0.1	T
177	377777607760771	3	1.4	50	0.1	6.0	LAM
1356	776377777760751	3	1.4	12	0.0	25.0	S
1827	777703600020771	3	1.4	6	0.0	50.0	Haarlem
1832	777773607760771	3	1.4	14	0.0	21.4	LAM
7	377777777760771	2	0.9	54	0.1	3.7	T
33	776177607760771	2	0.9	770	1.8	0.3	LAM
36	777737777720771	2	0.9	77	0.2	2.6	T
47	777777774020771	2	0.9	1021	2.4	0.2	Haarlem
49	777777777720731	2	0.9	115	0.3	1.7	Haarlem
60	777777607760731	2	0.9	178	0.4	1.1	LAM
95	777777607560731	2	0.9	30	0.1	6.7	LAM
125	000000007760731	2	0.9	55	0.1	3.6	unknown
737	777777607760571	2	0.9	11	0.0	18.2	LAM
753	477777607760771	2	0.9	16	0.0	12.5	LAM
828	377777607760731	2	0.9	20	0.0	10.0	LAM
1803	617777607760771	2	0.9	7	0.0	28.6	LAM
1	000000000003771	1	0.5	5610	13.2	0.0	Beijing
2	000000004020771	1	0.5	337	0.8	0.3	Haarlem
17	677737607760771	1	0.5	473	1.1	0.2	LAM
20	677777607760771	1	0.5	588	1.4	0.2	LAM
56	777737770000000	1	0.5	18	0.0	5.6	unknown
58	777777557760771	1	0.5	119	0.3	0.8	unknown
62	777777774020731	1	0.5	233	0.5	0.4	Haarlem
92	700076777760771	1	0.5	328	0.8	0.3	X
102	777703777760771	1	0.5	56	0.1	1.8	unknown
162	777777607760671	1	0.5	17	0.0	5.9	unknown
211	776137607760771	1	0.5	63	0.1	1.6	LAM
254	777760007760771	1	0.5	120	0.3	0.8	LAM
393	777757777760771	1	0.5	19	0.0	5.3	T
397	777777600000771	1	0.5	13	0.0	7.7	unknown
439	777376777760601	1	0.5	3	0.0	33.3	X
578	637777607760771	1	0.5	27	0.1	3.7	LAM
746	777777777520771	1	0.5	22	0.1	4.5	Haarlem
765	777760077760771	1	0.5	7	0.0	14.3	LAM
781	000000004020731	1	0.5	9	0.0	11.1	Haarlem
784	776377777760731	1	0.5	39	0.1	2.6	S
1063	776377777660771	1	0.5	24	0.1	4.2	S
1367	377737607760771	1	0.5	3	0.0	33.3	LAM
1610	763777607560771	1	0.5	5	0.0	20.0	LAM
1892	777777660000171	1	0.5	5	0.0	20.0	unknown
2644	177777777720771	1	0.5	3	0.0	33.3	Haarlem

**Table 2:** Characteristics of 15 orphan *M. tuberculosis *strains identified in Paraguay, 2003

Strain No.	Binary spoligotype (octal number)	RFLP	MIRUs description (MIT)	Lineage
Py63		Unique	213326153223 (unknown)	T
Py204		nd	124326153227 (71)	LAM
Py240		nd	nd	unknown
Py133		Unique	nd	Haarlem
Py149		nd	124327133226 (unknown)	LAM
Py244		nd	nd	LAM
Py201		Unique	223325153326 (unknown)	unknown
Py91		Unique	124326133223 (unknown)	LAM
Py21		Unique	124326153224 (140)	LAM
Py59		Unique	*323251*3322 (unknown)	unknown
Py95		Clustered	225325154323 (182)	Haarlem
Py31		nd	124326153225 (427)	LAM
Py93		Unique	nd	unknown
Py87		Clustered	233325153224 (218)	unknown
Py136		Unique	nd	unknown

The frequencies of major *M. tuberculosis *spoligotype families as defined in the SpolDB4 database [14] ranked in the following order: Latin-American & Mediterranean (LAM) 52.3% (115/220), Haarlem 18.2% (40/220), S clade 9.5% (21/220), an ill-defined T superfamily (not a clade *sensu stricto *as it is defined by default) 8.6% (19/220), X clade 0.9% (2/220) and Beijing clade 0.5% (1/220). The sole Beijing strain in the study was pansusceptible and had been obtained from a 47 year-old female South Korean immigrant living in Asuncion. Twenty-two (10.0 %) strains showed patterns that did not fit within any of the major clades described in SpolDB4.

### IS*6110 *RFLP analysis and epidemiological clustering

Patterns of the 165 isolates analyzed by IS*6110 *RFLP contained between four and 19 bands (mean ± SD: 11 ± 3). The sole strain showing an RFLP pattern with four bands belonged to the X lineage (the other strain of the X lineage in the study was not available for RFLP). One hundred and nineteen distinct RFLP patterns were observed. When only IS*6110 *RFLP was considered for clustering analysis, 65 isolates were grouped in 19 clusters of 2, 3, 5, 9 and 10 strains. When both RFLP and spoligotype were analyzed together, four RFLP clusters were further split off and five strains were classified as orphan (see Table 3).

**Table 3:** Characteristics of clusters identified by RFLP IS*6110 *and spoligotyping in Paraguay, 2003

**RFLP Cluster**	**Size**	**Spoligotype**		**Country region (n)**	**Likely link (n)**
				
		**SIT**	**Lineage**		
A*	9 (8)	2643	Haarlem	Metropolitan Area (7)	Tacumbu Jail inmates (6)
				Oriental (1)	-
B	2	49	Haarlem	Oriental (2)	Undetermined
C*	10 (8)	34	S	Metropolitan Area (2)	Undetermined
				Oriental (6)	Neighbor towns (5)
D	3	34	S	Chaco (3)	Aboriginal community (3)
E	2	34	S	Oriental (2)	Undetermined
F	2	1356	S	Metropolitan Area (1)	-
				Oriental (1)	-
G	5	42	LAM	Metropolitan Area (4)	Household (3)
				Chaco (1)	-
H	3	42	LAM	Metropolitan Area (1)	Tacumbu Jail
				Oriental (2)	Undetermined
I	2	42	LAM	Metropolitan area (2)	Tacumbu Jail (2)
J*	2 (0)	42 2648	LAM	Oriental (2)	Same town (2)
	(3)	42	LAM	Metropolitan Area (1)	-
K*	5			Oriental (2)	Same department (2)
	(2)	753	LAM	Oriental (2)	Undetermined
L	3	177	LAM	Oriental (3)	Same community (2)
M	3	391	LAM	Metropolitan Area (2)	Undetermined
				Chaco (1)	-
N	2	391	LAM	Chaco (2)	Aboriginal communities (2)
O	3	2647	LAM	Oriental (2)	Undetermined
				Chaco (1)	-
P	2	2650	LAM	Oriental (2)	Same town (2)
Q	2	93	LAM	Chaco (2)	Aboriginal community (2)
R	3	52	T	Chaco (3)	Aboriginal communities (3)
S	2	521	T	Chaco (2)	Aboriginal communities (2)

* RFLP clusters marked with an asterisk contain one or more strains with different spoligopattern(s) and numbers in brackets indicate cluster size after subtraction of strains with different spoligopattern.

Concordant RFLP and spoligotype clustering involved 15/30 (50%), 20/54 (37%) and 25/81 (31%) strains from the Chaco, the metropolitan area and the Oriental Region, respectively. No association was found between clustering and gender (45/122 among males vs. 16/43 among females, p = 0.88), age (42/99 < 50 yrs vs. 20/66 ≥ 50 yrs, p = 1.16), treatment history (10/29 previously treated vs. 47/136 never treated, p = 0.70) or drug resistance (7/29 with any drug resistance vs. 54/136 with no resistance, p = 0.17). Demographic evidence was consistent with transmission in particular settings such as the Tacumbu jail in Asuncion, indigenous communities in the Chaco, and a single household in the metropolitan area.

Strains harboring the SIT34 of the S clade grouped in the RFLP cluster C together with two other strains harboring rare spoligotypes: an orphan strain (Py87) and a strain of the new SIT 2653 (Py51). These two novel spoligopatterns could be visually considered to be derived from the SIT 34 associated to this RFLP cluster (Figure 1).

Similarly, identical or closely-related RFLP patterns were exhibited by strains classified within each of five new shared spoligotypes identified exclusively in Paraguay. These are SITs 2643 (cluster A), 2647 (cluster O), 2650 (cluster P), 2645 and 2654 (Figure 1). In cluster A, IS*6110 *RFLP grouped strains of the SIT2643 together with one strain harboring an orphan spoligotype different from but closely related to SIT 2643. Most strains in this cluster (cluster A) had been isolated from inmates in the Tacumbu jail. Apart from these two orphan strains grouped by RFLP in clusters A and C, the remaining 13 strains with orphan spoligotypes did not match with any of approximately 2000 RFLP patterns contained in the database at the Malbran Institute, which gathers patterns from strains isolated in Argentina and other South American countries.

### MIRU-VNTR analysis of new spoligopatterns

Results of MIRU analysis of 30 selected strains with either new SITs or orphan spoligotypes are summarized in Table 2 and Figure 1. MIRU typing confirmed the clonal nature of two of the five newly described SITs restricted so far to Paraguay: SIT 2650-MIT 427 of the LAM lineage and SIT 2643-MIT 182 of the Haarlem lineage. This latter clone is hereafter named "Tacumbu" after the men's correctional facility in Asuncion where it was most frequently found; it included another strain in the same RFLP cluster with an orphan but related spoligopattern (Py95) isolated from another inmate in the same jail (Figure 1).

## Discussion

We present herein a first insight into the biodiversity of the *M. tuberculosis *epidemic in Paraguay. At the same time, we provide evidence of the suitability of IS*6110 *RFLP as a genotyping tool for epidemiological studies in the country, either as a stand-alone tool or, still better, in combination with spoligotyping.

Even though the characteristics of the study sample favored phylogenetic rather than epidemiological analysis, RFLP analysis by itself produced a concise picture of TB transmission patterns in Paraguay. In particular, it served to identify transmission in specific settings such as indigenous communities in the Chaco and the Tacumbu jail in Asuncion. The percentage of clustering in different regions was congruent with the respective regional incidence rates, i.e. highest in the Chaco, intermediate in the densely populated metropolitan area, and lowest in the rest of the Oriental Region.

We did not find correlation of clustering with previous TB treatment or drug resistance, suggesting that in Paraguay most patients develop drug resistant TB individually through selective pressure imposed by poorly constructed or inadequately supervised treatment regimens rather than through transmission of resistant strains. The age and gender composition of patients in our sample reflects the predominance of TB among young male people in Paraguay. This disease distribution is common to many low income settings worldwide and could be attributed to socioeconomic and cultural barriers in the access to health care [15]. In the present study, however, we failed to find association of clustering with gender or age, probably due to the small size of the sample and the short sampling period. In this sense, we are aware of the fact that genotype clustering analysis underestimates transmission and that this bias is inversely proportional to length of time and size of the sample [16].

The relative frequencies of major *M. tuberculosis *spoligotype families were roughly in range with the overall frequencies described for countries in the South American region [14,17-20]. The largely predominant LAM lineage, identified in more than half of the strains in our study, was ubiquitous in the country. Both its high degree of dissemination and its preponderance among the new (shared as well as orphan) patterns are manifestations of the current adaptative evolution of the LAM lineage in Paraguay. Second in frequency, the Haarlem clade was found mainly in the metropolitan area. Third in frequency, the S clade was found predominantly in the Oriental Region. The strains classified within the ill-defined T family were widely distributed within the country. The IS*6110 *low-banding pattern X genotype family was rather unusual and the Beijing genotype was absent in the native population of our study. The sole Beijing strain identified in the study was isolated from an East Asian immigrant who most probably acquired her infection prior to arriving in Paraguay.

An example of congruence between phylogenenetic and epidemiological findings is the fact that the four most prevalent SITs in our study accounted for two thirds of the RFLP/spoligotyping clustered cases, including cases with epidemiological links (e.g., inmates in Tacumbu jail, cases in aboriginal communities, household contacts). These were SIT42 of the LAM9 clade, SIT391 of the LAM4 clade, SIT34 of the S clade and the newly-created SIT2643 of the Haarlem clade. As shown by their active ongoing transmission, these SITs could be regarded as highly successful *M. tuberculosis *genotypes in Paraguay. Interestingly, two of these *M. tuberculosis *sub-lineages seem to have a restricted geographical distribution: SIT391 has been so far found only in Brazil (n = 2) and Paraguay (n = 21) and the newly created SIT2643 (n = 13) is restricted to Paraguay alone. IS*6110 *RFLP and MIRU typing confirmed the clonality of this latter phylogeographically specific genotype, which is hereby designated as "Tacumbu" genotype. Indeed, this clone is completely new as it created not only a new SIT (SIT2643) but also matched a rare MIT (MIT182) in the database within the Haarlem clade.

Although different mutation mechanisms may converge into identical spoligopatterns, the main force driving variation in the DR region appears to be deletion of single or contiguous direct variable repeat sequences [21]. Our data supports this kind of evolution for some actively trasmitting clones, which may represent emerging genotypes in Paraguay. For example, the main chain of transmission in Tacumbu jail included an orphan strain that shared identical MIT and RFLP with other strains in the cluster and harbored a spoligotype that could well have evolved from the same Tacumbu SIT through deletion of two contiguous spacers. Likewise, SIT391, endemic in Paraguay (n:21), has probably evolved to the new SIT2650 (n = 2) by the loss of one spacer. Similar deletions in the DR region could have also occurred in two rare strains harboring MIT218 and fitting within the largest RFLP cluster together with 8 strains of the S clade.

In addition to rapid evolution of some predominant strains, spoligotyping revealed the geographical specificity of a number of *M. tuberculosis *strains in our study. Similar to findings reported in Venezuela, Brazil and Suriname [17,18,22] a number of spoligopatterns in our study were unique among more than 45,000 strains recorded in the SpolDB4. Most of these strains proved further their singularity when tested by RFLP and/or MIRUs. Moreover, almost one in ten strains did not fit into any of the major *M. tuberculosis *phylogenetic clades described in the updated database and one in five spoligopatterns in the study were rarely or never described outside Paraguay.

## Conclusion

Paraguay's current TB epidemic seems to consist of a wide diversity of sublineages among which a few LAM, Haarlem and S genotypes prevail and are evolving actively. The lineages of tubercle bacilli currently thriving in this rather secluded South American niche may reflect the local host-pathogen adaptation of strains introduced into the country during past migrations from European countries [23].

## Methods

### Clinical isolates

The 220 *M. tuberculosis *strains examined in this cross-sectional study were isolated from the same number of patients with pulmonary TB and positive acid fast bacilli smear examination recruited consecutively during the national survey of drug resistance carried out in Paraguay during 2003. These strains represented 77% of the 286 strains composing the drug resistance survey, which was designed applying the cluster sampling procedure. The 220 strains of the survey available for genotyping did not differ from those lost to genotyping with respect to drug susceptibility, geographical origin and patient characteristics. Data concerning patient gender, age, geographic origin, date of diagnosis and previous history of TB were collected from the survey questionnaire. Culture was performed on Löwenstein-Jensen slants in five laboratories of the national TB network. Species identification and susceptibility testing to first line anti-TB drugs were performed at the Central Public Health Laboratory of the Ministry of Health using conventional biochemical tests and the standard proportion method on Löwenstein-Jensen slants [24].

### Genotyping

Chromosomal DNA was prepared by the cetyl-trimethyl ammonium bromide method from heat inactivated bacilli suspensions [25]. Spoligotyping was performed on all 220 isolates by reverse hybridization as described previously [8]. *IS6110 *restriction fragment length polymorphism (RFLP) was performed according to the standard protocol [26] on the 165 specimens that yielded a sufficient amount of unbroken DNA to successfully perform the protocol. Probe labeling, in the case of RFLP, and detection of hybridizing DNA, in both RFLP and spoligotyping, was done by enhanced chemiluminiscence (ECL kit, Amersham, Little Chalfont, England) followed by exposure to X-ray film (Hyperfilm ECL, Amersham).

The number of repeats of 12 MIRUs (MIRU No. 2, 4, 10, 16, 20, 23, 24, 26, 27, 31, 39 and 40) was investigated on selected isolates by amplification and visual assessment of amplicon size as previously described [27]. PCR mixtures consisted of 0.5 μM (each) dNTP (Qbiogene), 2 mM MgCl_2 _(Amersham), 10% DMSO, 1 × recombinant Taq buffer mix (Amersham), 0.5 U rTaq (Amersham), 0.3 μM each primer and 1 μl DNA. The final volume of each reaction was 30 μl. Samples were amplified in a GeneAmp PCR system 9600 (Perkin Elmer-Applied Biosystems-Basel, Switzerland) by using an amplification profile of 10 min at 94°C, 35 cycles of 30 s at 94°C, 60s at 60°C and 2 min at 72°C. DNA fragments were separated by electrophoresis on a 1.5% agarose gel (Invitrogen) and the number of MIRU copies corresponding to the respective bands sizes was then calculated. The tables used for MIRU-VNTR allele scoring are available at [28].

### Computer analysis

Digitalized images of autoradiographs were submitted to computer analysis using the software Bionumerics version 4.0 (Applied Math, Sint-Martens-Latem, Belgium), as described previously [29]. RFLP intra- and inter-experiment normalization was performed using strain *Mt14323 *DNA as an external marker. Similarity among banding patterns was calculated using the Dice coefficient with 1% tolerance and 1% optimization. Clustering analysis was performed by the unpaired weight of mathematical averaged method. Clusters were defined as groups of patients infected with *M. tuberculosis *strains showing identical RFLP and spoligopatterns.

Spoligotypes were entered in an updated version of the SpolDB4 database [14]; the unpublished in-house updated version is alternatively termed as SITVIT2 database.

## Authors' contributions

NC carried out the molecular genetic studies, analyzed the data and contributed to drafting the manuscript. BL participated in genotyping studies, analyzed the data and provided suggestions during manuscript preparation. TZ carried out molecular genetic studies, participated in the identification and designation of the SITs and conducted the bioinformatic analysis. MC assisted with data entry and conducted the bioinformatic analysis. CD contributed to the molecular genetic studies, data entry and the review of the manuscript. She also provided technical help in the conservation and management of the strains. GR conceived the study, participated in its design, coordinated the investigation, and provided critical comments to the manuscript. NJR carried out mycobacteriological diagnostics, isolation, identification and drug susceptibility testing of clinical isolates, and provided information about the clinical isolates for molecular study. JCJ participated in the design and carried out the survey of anti-tuberculosis drug-resistance, analyzed the data, and provided the clinical isolates for molecular study. LB participated in the design of the study, and provided critical comments for the manuscript. NR carried out the phylogeny reconstruction studies, participated in the identification and designation of the SITs and also helped in the draft of the manuscript. VR conceived the study and the methodology, coordinated the investigation and wrote the manuscript.
